# Behavioural Responses of Beef Cattle to Hot Conditions

**DOI:** 10.3390/ani14162444

**Published:** 2024-08-22

**Authors:** Musadiq Idris, Megan Sullivan, John B. Gaughan, Clive J. C. Phillips

**Affiliations:** 1 Faculty of Veterinary and Animal Sciences, The Islamia University of Bahawalpur, Bahawalpur 63100, Punjab, Pakistan; 2 School of Agriculture and Food Sustainability, Gatton Campus, The University of Queensland, Gatton, QLD 4343, Australia; megan.sullivan12@outlook.com (M.S.); j.gaughan@uq.edu.au (J.B.G.); 3 Curtin University Sustainability Policy Institute, Faculty of Humanities, Curtin University, Perth, WA 6845, Australia; clive.phillips@curtin.edu.au; 4 Institute of Veterinary Medicine and Animal Science, Estonia University of Life Sciences, 51014 Tartu, Estonia

**Keywords:** behavioural response, beef cattle, heat load, heat stress, high temperature, stepping

## Abstract

**Simple Summary:**

A better understanding of cattle behavioural responses during high environmental temperatures could be beneficial for the diagnosis of heat loads. In the current study, 24 Black Angus steers fed a finisher diet based on cereal grain or a substituted diet (8% of the grain replaced by an isoenergetic amount of lucerne hay) were exposed to hot conditions, and changes in their behavioural and physiological dynamics were assessed. Compared to the recovery period, hot conditions increased respiration rate and were associated with increased stepping behaviour, especially by left limbs. Cattle also increased the orientation of their heads downward, their ears backward, and their tail vertically or raised, and they reduced eating, grooming, and scratching during the heat load period. Changes in head, ear and tail position, breathing changes persisted during the heat load period, suggesting that these behaviours have diagnostic value. Cattle that were fed a diet based on the cereal grain stood for longer and were more likely to hold their ears backward and tail vertically, whereas those on the substituted diet were more likely to display axial ears and hold their head in a neutral position. We conclude that cattle have subtle changes in behaviour in response to hot conditions, which may be of value for diagnostic purposes.

**Abstract:**

Cattle are increasingly exposed to hot temperatures as a result of climate change, and a better understanding of behavioural responses could be beneficial for the diagnosis of heat loads. The changes in the positioning of key body parts, feeding behaviour, body maintenance, and respiratory dynamics were assessed in 24 Black Angus steers individually exposed to hot conditions and fed a finisher diet based on cereal grain or a substituted diet (8% of the grain replaced by an isoenergetic amount of lucerne hay). Increased respiration rate during the heat load period, compared to the recovery period, was associated with increased stepping, especially by left limbs. Cattle also reduced eating, grooming, and scratching during the heat load period. The lowered head, backward ear, vertical or raised tail, and increased respiration rate and panting persisted in cattle during the heat load period. Cattle on the cereal grain diet stood for longer and were more likely to hold their ears backward and tail vertical than those on the substituted diet. We conclude that these behaviours could be used to detect animals that are most affected and that changing from a cereal-based diet to a substituted diet containing a higher amount of fibre, such as lucerne hay, can reduce hyperthermic behavioural responses to a heat load.

## 1. Introduction

In hot climates, rapidly growing beef cattle are particularly susceptible to heat load because of their high metabolic rate and level of nutrition [1,2]. In some regions, the feedlot industry experiences significant economic losses in health and production, as well as mortality, due to the excessive heat throughout the summer period [1]. Adaptation and acclimation to climatic conditions are critical for sustainable livestock production. Thermal tolerance is considered an adaptive response of cattle [3]. Cattle can potentially invoke both behavioural and physiological responses to attain a thermal balance during excessive heat load conditions [4]. Physiological responses include increases in core body temperature, respiratory exchange, and sweating [5,6], as well as changes in liver, kidney, and hypothalamus functions [7], all mediated by endocrine responses, principally glucocorticoids, antidiuretic hormones (ADHs), growth hormones (GHs), thyroxine, prolactin (PRL), and aldosterone [8]. The behavioural responses of cattle to a heat load condition are less well defined but include increased water consumption, reduced feed consumption, respiration and panting behaviour, shade seeking, water splashing, crowding at the water trough, and clustering [9,10].

An accelerated inspiratory/expiratory exchange (respiration rate, RR) and panting score (PS) can be early signs of increasing heat load as heat is lost in evaporative transpiration from the body surface. Hahn et al. [11] reported an increase in respiration rate by 4.3 breath/min per °C rise above the baseline respiration rate (60 breaths /min) at a threshold temperature of 21.3 °C. Cattle increase water consumption under heat load, with Black Angus steers during hot treatment (daily maximum ambient temperature (TA): 33 °C) drinking 40.7 ± 0.96 L/d, compared to 30.5 ± 0.85 L/d drunk by cattle in a thermoneutral (TA; 19–23 °C) environment [12]. Feed intake declines, which diminishes heat output from feed digestion [13]. Cattle also prefer to stand rather than lie down so that they can increase the body surfaces available to lose heat by evapotranspiration, especially through the skin on the underside of the animal’s torso, which is less protected by a covering of hair, thus exposing the skin to cooling airflow [14]. To reduce the radiant heat load from the sun, there is an increase in shade-seeking behaviour [15]. Other behavioural changes include reduced or no rumination and, where possible, wallowing [16]. 

Behavioural responses help cattle to adjust to environmental conditions and thereby assist in the maintenance of homeostasis [9,17]. However, there may also be psychological elements in the response, such as movement responses common to many stressors, which increase heat production and airflow around the body. There may be leg movement (stepping) and ear, head, and tail movement. These body movements have been investigated in response to a variety of stressors but not a heat load [18,19,20]. Further studies are needed to assess behavioural responses under conditions that simulate those experienced in feedlots in Queensland, Australia. Therefore, the objective was to assess the evidence for heat load responses during closely controlled hot conditions, with the aim of improving the welfare of animals [16], measured through the changes in behavioural responses as a non-invasive tool, and their relation to other variables known to be associated with the response of livestock to heat stress–respiratory rate, panting score, standing, lying, feeding, drinking duration, and other behaviours. Further, it was investigated whether a substituted diet (grain substituted with forage) would affect the behavioural responses to heat stress, measured through changes in behavioural responses.

## 2. Materials and Methods

### 2.1. Study Design

Ethical approval for this study was obtained from the University of Queensland Animal Ethics Committee (SAFS/460/16). Briefly, twenty-four yearling Black Angus steers with a mean initial non-fasted body weight of 493 ± 6.8 kg were procured from a commercial property in Armidale, New South Wales, Australia and transferred to the experimental facility at the University of Queensland, Gatton, Australia-QASP in the southern hemisphere summer period. The animals were randomly divided into two cohorts of 12 animals (a cereal-based finisher diet and a substituted diet) to be run separately through the climate-controlled facility, which only had a capacity for this number of animals. The first cohort was fed a standard feedlot ration based on cereal grains (finisher diet), and the second received a diet in which 8% of the grains were replaced by an isoenergetic amount of lucerne hay (substituted diet). The complete details of the animals and experimental treatments can be found in the paper by Idris et al. [21].

Briefly, the animals were kept in a climate-controlled facility for 18 days, where they were exposed to an initial thermoneutral period (TN; d 3 + 4) for an acclimatisation period (ACC), followed by a transition phase to hot conditions (TP1; d 5), a hot period (HOT; d 6–12), a transition phase to the recovery period (TP2; d 13), and a recovery thermoneutral period (d 14–17), as described in Table 1. In the TN and recovery periods, the ambient dry-bulb temperature (T_A_) and relative humidity (RH) in the climate-controlled facility were maintained at 20 °C and 65%, respectively. The climatic conditions inside the climate-controlled facility were centrally controlled through programmed air circulation. In the HOT period, T_A_ and RH varied daily from 700 h to reach a maximum at 1100 h, which was maintained until 1600 h and then decreased hourly to the daily minimum T_A_ and RH at 2000 h. To simulate a typical heat wave in Queensland, the ambient temperature declined over the HOT period from d 6 to d 12 before cattle entered TP2 and the recovery period (Table 1).

### 2.2. Animal Housing

The details of the feedlot pens and climate-controlled facility have also been described in detail by Idris [22] and Idris et al. [21]. Briefly, in the initial feedlot phase, each cohort of animals was kept in a feedlot pen of 162 m^2^ (27 × 6 m), with an east–west alignment. The animals were then randomly assigned to individual pens (each 10 × 3.4 m) in an outdoor facility and then housed in a climate-controlled facility where they were assigned to individual pens. The climatic conditions inside each pen were centrally controlled. The climate-controlled facility was equipped with cameras (K-guard CW214H; New Taipei City, Taiwan), two over each pen attached to a digital video recorder (LG, XQ-L900H; Yeouido-dong, Seoul, South Korea), for the surveillance of the animals.

### 2.3. Animal Management

The steers were immunised following a similar regime to that outlined by Sullivan et al. [23]. Briefly, in the feedlot, the animals in the first cohort were provided with a starter diet of concentrates for the first 8 d, followed by an intermediate diet for 6 d before transitioning to a finisher diet over 3 d, which was then fed until the end of the trial (Table 2). Due to an adverse heat stress response of some animals in the first cohort, the second cohort was fed the substituted diet from the second day of the HOT period and was transitioned back to a finisher diet over four days during the thermoneutral recovery period. Experimental animals were fed their diet on a dry-matter basis at 2.5% of their body weight. Individually housed cattle were fed their diet at 2.5% of their body weight on a DM basis, with refusals removed and weighed each morning prior to the provision of 50% of the ration at 900 h and the remainder at 1300 h. Feed dry matter content was determined by oven drying. The animals were provided with ad libitum water during this study, and water consumption was recorded at the time of each observation using endurance multi-jet turbine water meters (RMC Zenner, Eagle Farm, Australia).

### 2.4. Behavioural Observations

#### 2.4.1. Visual Observations

Respiratory behaviour and other key behavioural observations, including changes in the positioning of key body parts, feeding behaviour, drinking behaviour, and body maintenance, were recorded every 2 h from 600 to 1800 h during the TN and recovery periods and every hour over each 24 h during the HOT period. The data from the observational behavioural study were recorded as instantaneous and or scan samples [24] by a team of five trained observers, who also recorded the respiration rate and panting score (PS), with only one observer recording measurements at any one time (Table 3). The respiration rate (RR) was determined by recording the time taken for the animal to take ten breaths observed from flank movements, converted into breaths per minute. The panting scores (PSs) of animals were visually scored using a modified scale of 0 to 4.5, where PS 0 indicates no heat stress and PS 4.5 represents a severely heat-stressed animal [2,14,25]. The number of chews while eating was determined by counting chews for one minute at the time of the morning feed.

#### 2.4.2. Observations from Video Recordings

From the video recordings, standing, lying, stepping of each limb, eating, ruminating, grooming, and scratching were continuously observed using the event-logging software BORIS v. 6.0.4 [26] for 5-min intervals every h for 24 h on d 3 (TN), d 6–12 (HOT), and d 14–16 (recovery) (Table 3). The right/left limb ratio, (right and left limb stepping relative to each other) and front/back limb ratio (front and back limb stepping relative to each other) were also calculated from the recorded stepping of each limb. Head, ear, and tail positions were also observed at 5-min intervals every h for 24 h. Not all behaviours could always be observed: accurate recording of ear position was not possible when the animal was ruminating, eating, drinking, scratching, or grooming; recording of the head position was not possible during eating, drinking, grooming, or scratching, and recording of the tail position was not possible during defecation, urination, eating, drinking, grooming, or scratching. 

#### 2.4.3. Post-Experiment Behavioural Observations

The post-experiment behaviour (rebound behaviour) that comprised grooming, scratching, eating, drinking, rumination, standing, and lying, including head, tail, and ear orientations of the cattle, was observed at 5-min intervals from 900 h to 1700 h daily for the first two days after they had returned to the experimental feedlot from the climate-controlled facility.

### 2.5. Climatic Data 

The ambient temperature and humidity inside the climate-controlled facility were maintained through a programmed cyclic air-conditioning system. A temperature–humidity index (*THI*) was calculated using the following equation, adapted from Thom [27]:

*THI* = (0.8 × *T_A_*) + {[(*RH*/100) *×* (*T_A_* − 14.4)} + 46.4] (1)

where *RH* = relative humidity in % and *T_A_* = ambient temperature in °C. 

### 2.6. Statistical Analyses

Four animals (two from each cohort) were removed from the experiment due to their adverse response to the heat load at the start of the HOT period: two in the first cohort and two in the second cohort. Therefore, in each dietary cohort, the data obtained from 10 steers were analysed using the statistical software Minitab 17 (Minitab^®^ 17.3.1 Inc., Chicago, IL, USA) and Minitab 18 (Minitab^®^ 18.1 Inc., Chicago, IL, USA) for Windows.

Two separate models were used to describe the data, the first being a simple comparison between the TN period and the HOT period, which included animal ID as a random factor and with the following fixed factors: cohort (*D*; finisher and substituted diets) and treatment period [*P*; HOT and TN], as well as the treatment period × diet interaction. Only Period and *D* × *P* interactions are presented as diet did not change until the HOT period. The second model was a mixed effects model, which was used to determine the biological responses of feedlot cattle for the two cohorts through a comparison of behaviour in the HOT period to that in the recovery period. The model comprised the same random and fixed factors as the first one, with the addition of day as a fixed factor nested within the treatment, and the data from the TN period were used as a covariate (*Cov*) against the treatment period [*P*; HOT and recovery], as well as the following interactions: diet x treatment period and diet x day. The equation used for the analysis is as follows:

*Y_B_* = *µ + D + P + d*(*P*) *+ ID +* (*D × P*) *+* (*D × d*(*P*)) *+ Cov + e* (2)

where $Y_{B}$ is the expected value for biological response variables, *μ* is the expected mean value for response variables when input variables = zero (the factors are as described above), and e is the random error associated with experimental observations.

In both models, pairwise comparisons between treatment means were performed using Fisher’s test. Logarithm transformations (log_10_ + 1) were made for some variables in order to achieve an approximate normal distribution of the residuals. When the proportion of zeros was more than 50% and a linear model produced residuals that were not normally distributed, the data were dichotomised into a binary format according to whether the cattle did or did not perform the behaviour each day and were analysed by binary logistic regression using a logit model. Raised head position, downward ear position, and raised tail were analysed in this way. There were few behavioural events of raised ears and tucked tail; so, they were analysed using a chi-square goodness-of-fit test to estimate daily behavioural counts for each animal/day during each experimental period.

To determine whether the responses of individual animals to the HOT period were related to their behaviour in the feedlot after the recovery period, Spearman’s rank correlation coefficients (r_s_) with a two-tailed level of significance (*p* < 0.05) were determined. The Spearman’s rank correlation explains the relationship between their behaviour in the feedlot and the difference between the behaviour of cattle during the HOT period and that in the first thermoneutral period (HOT − TN), i.e., the cattle that reacted most to the high temperatures. The Benjamini–Hochberg procedure was used to decrease the false discovery rate, with a critical value for a false discovery rate of 0.25 [28]. A principal component analysis (PCA) was performed using the Minitab statistical package (Minitab^®^ 17.3.1 Inc., Chicago, IL, USA) in order to understand the relationships between behavioural variation estimates.

## 3. Results

### 3.1. Stepping, Standing, and Lying

The behavioural responses of the feedlot cattle (n = 20) exposed to high temperatures (HOT) compared to the TN are presented in Table 4. The stepping rates of all four limbs and standing time were greater, and lying was decreased in the HOT period compared to the TN period. There was also a significant reduction in the R/L limb ratio, (an increase in left limb stepping relative to right limb stepping), and similarly, there was a reduction in the F/B limb ratio (more back limb stepping than front limb stepping; F/B ratio < 1) during the HOT period compared to the TN period.

The behavioural responses of the cattle when exposed to the HOT and recovery periods are shown in Table 5. The stepping rates of all four limbs, standing time, and back limb stepping relative to front limb stepping were greater in the HOT period than in the recovery period. Lying time did not differ between the recovery period and the HOT period.

**Figure 1:**
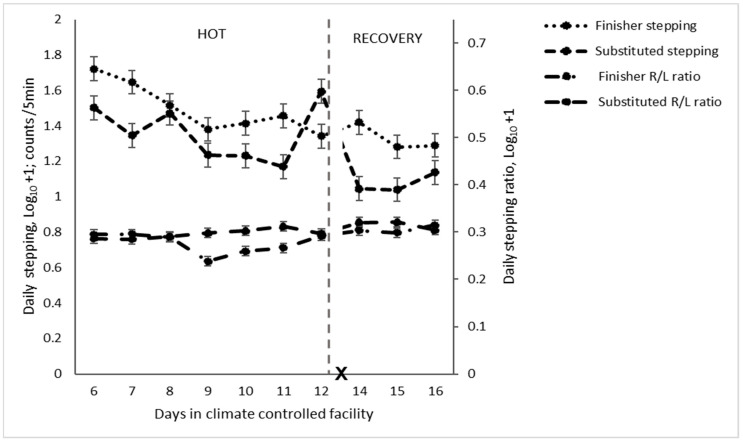
Daily stepping behaviour of feedlot cattle on different diets exposed to high temperatures (HOT) or a recovery period (R/L = right/left limb stepping ratio and X = d 13 not analysed).

**Figure 2:**
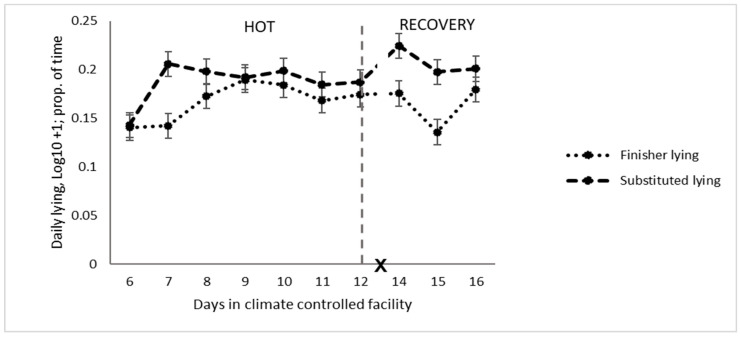
Daily lying pattern of feedlot cattle on different diets exposed to high temperatures (HOT) or a recovery period (X: d 13 not analysed).

Stepping was greater for cattle on the finisher diet than the substituted diet on all days, except on the last day of HOT when the substituted diet had a large increase in stepping (Table 4 and Table 5 and Figure 1). There was more left limb stepping in the finisher diet than the substituted diet relative to right limb stepping but only in the second three days of the HOT period. There was relatively more back limb stepping relative to front limb stepping in the finisher diet compared to the substituted diet. Standing time was greater in the cattle when fed the finisher diet, and lying was increased for cattle fed the substituted diet (Table 4 and Table 5), which was most pronounced on day 2 of HOT (*p* = 0.03, Figure 2).

### 3.2. Ears

Ears were held facing backward more often in the HOT than in the TN (Table 4) and recovery periods (Table 5). Ears were more axial and forward in the TN (Table 4) and recovery periods (Table 5) than in the HOT period. In the analysis of ears facing downward, which was by binary logistic regression, more cattle (80%) were observed to hold their ears downward at least once a day in TN compared to the HOT (42%) (OR 0.066; CI 0.013–0.33) and recovery periods (28%) (OR 0.0415; CI 0.008–0.22; *p* < 0.001). No significant differences in the raised ear position were observed during these three different periods (Chi-square, 0.05; *p* = 0.97).

**Figure 3:**
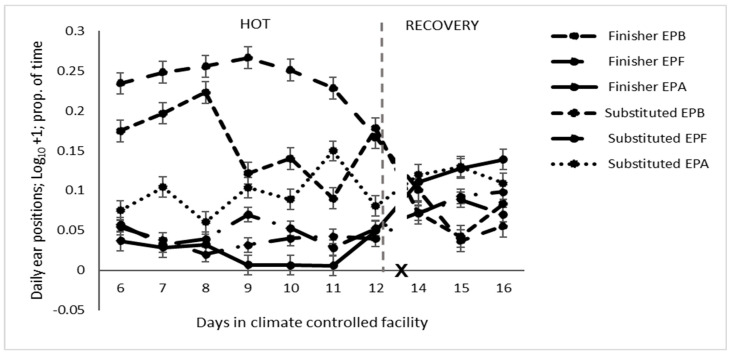
Daily ear positions of feedlot cattle on different diets exposed to high temperatures (HOT) or a recovery period (EPB: ear backward, EPF: ear forward, EPA: ear axial, and X: d 13 not analysed).

Ears in the HOT period were backward more in the finisher diet than the substituted diet, particularly during the second three days (Table 4 and Figure 3). In the HOT period, axial ears gradually increased in the substituted diet, whereas in the finisher diet, they were very rarely in this position throughout the period (Figure 3).

### 3.3. Head

**Figure 4:**
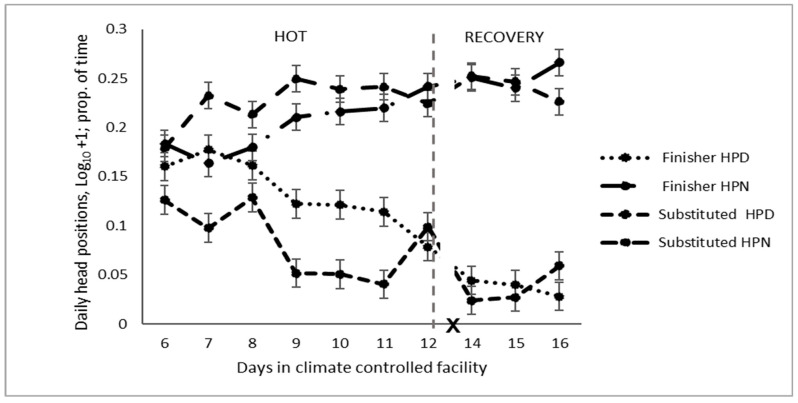
Daily pattern of head posture of feedlot cattle on different diets exposed to high temperatures (HOT) or a recovery period (HPD: head downward, HPN: head neutral, and X: d 13 not analysed).

A downward head position was more commonly observed for cattle in the HOT period than in the TN (Table 4) and recovery periods (Table 5). The difference in the lowered head position was most evident in the last three days of the HOT period (Figure 4). Conversely, holding their head in the neutral position was reduced in the HOT period compared to the TN (Table 4) and recovery periods (Table 5). No difference in the number of cattle with a raised head position was observed (Chi-square, 0.14; *p* = 0.93).

A downward head position was more commonly observed for cattle fed the finisher diet than those fed the substituted diet (Table 5 and Figure 4). Holding the head in the neutral position was reduced in the cattle on the finisher diet.

### 3.4. Tail

Cattle held their tails more frequently in a vertical position in the HOT period than in the TN period, but no difference in tail swishing was observed (Table 4). Cattle also held their tails more frequently in a vertical position, and there was less tail swishing in the HOT period than in the recovery period (Table 5).

In the HOT period, more cattle were observed to have their tails raised at least once a day (29%) compared to the recovery period (15%, OR 0.3430; CI 0.14–0.85; *p* = 0.05) and the TN period (25%, OR 0.806; CI 0.21–3.07). No difference in the tucked tail position was observed during different treatment periods (Chi-square, 2.08; *p* = 0.35). Cattle held their tail more frequently in a vertical position when on the finisher diet, with no difference observed in tail swishing between different dietary cohorts.

### 3.5. Oral Behaviours

Grooming and scratching were greatly reduced in the HOT period compared to the TN period (Table 4). Rumination time was greatly reduced and the chewing rate slightly was reduced during the HOT period compared to the TN period (Table 4). Eating time was approximately halved, and similarly, DMI was greatly reduced in the HOT period compared to the TN period (Table 5). Respiration rate and panting score approximately doubled during the HOT period compared to the TN period (Table 4).

Compared to the recovery period, grooming was reduced in the HOT period (Table 5). Scratching was rare during the HOT period but increased markedly during the recovery period (Table 5). Rumination time was greatly reduced during the HOT period compared to the recovery period (Table 5). Eating time was approximately halved, chewing rate slightly reduced, and DMI was reduced by 18–20% in the HOT period compared to the recovery period (Table 5). Respiration rate and panting score were approximately doubled during the HOT period compared to the recovery period (Table 5).

**Figure 5:**
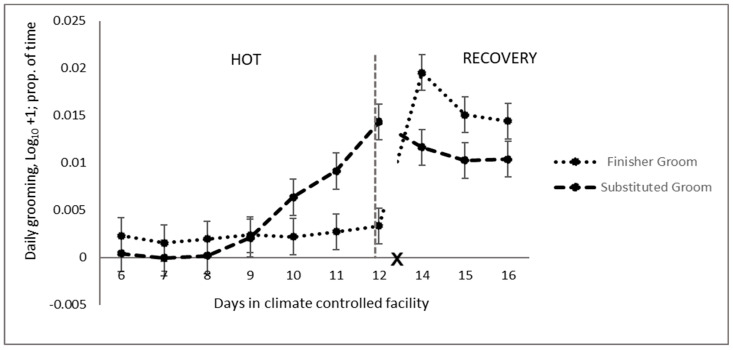
Daily grooming pattern of feedlot cattle on finisher or substituted diets during the high-temperature (HOT) and recovery periods (X: d 13 not analysed).

**Figure 6:**
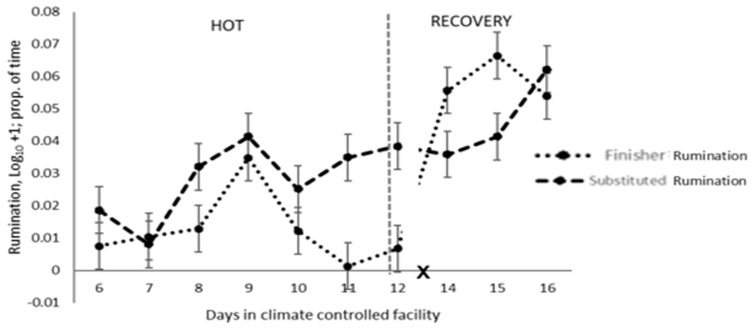
Daily rumination pattern of feedlot cattle on finisher or substituted diets during the high-temperature (HOT) and recovery periods (X: d 13 not analysed).

Overall, grooming was not different in the HOT period for substituted and finisher dietary cohorts (Table 5). However, grooming was greater in the animals receiving a substituted diet in the last three days of the HOT period (Figure 5), after which there was no difference in both dietary cohorts on d 16, with the completion of the dietary transition from the substituted diet to the finisher diet in these animals. No significant difference was observed in daily scratching activity in the animals on different diets (Table 5). Rumination time was greatly reduced in the animals on the finisher diet, principally in the last two days of the HOT period (Figure 6). In the recovery period, an effect was principally observed during the feed transition for cohort 2 (substituted diet), which was completed by d 16.

**Figure 7:**
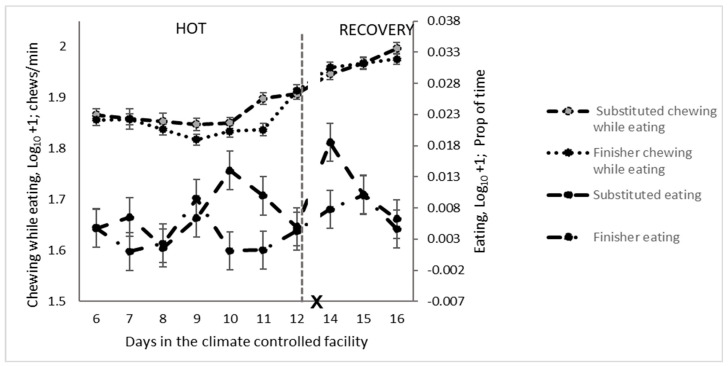
Daily eating and chewing-while-eating patterns of feedlot cattle on finisher or substituted diets during the high-temperature (HOT) and recovery periods (X: d 13 not analysed).

**Figure 8:**
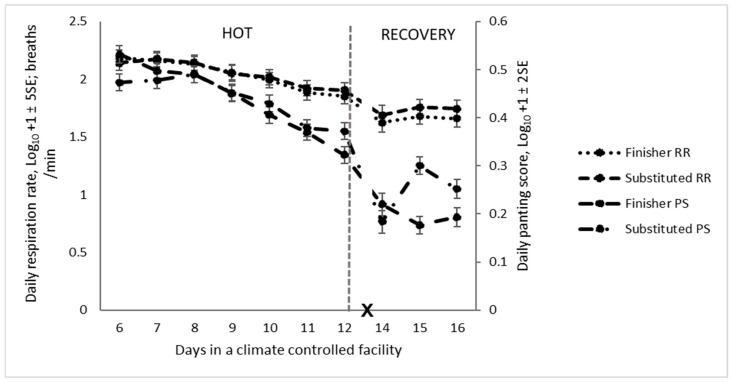
Daily respiratory dynamics (RR: respiration rate and PS: panting score) of feedlot cattle on finisher or substituted diets during the high-temperature (HOT) and recovery periods (X: d 13 not analysed) (RR: respiration rate, PS: panting score, and X: d 13 not analysed).

The eating time and chewing rate were higher in the last two days of the HOT period for cattle receiving the substituted diet (Figure 7). The respiration rate and panting score were not affected by diet during the HOT period; however, in the recovery period, they were both greater for cattle on the substituted diet (Table 5), particularly on d 15 and 16 (Figure 8).

### 3.6. Behaviour Correlations

**Figure 9:**
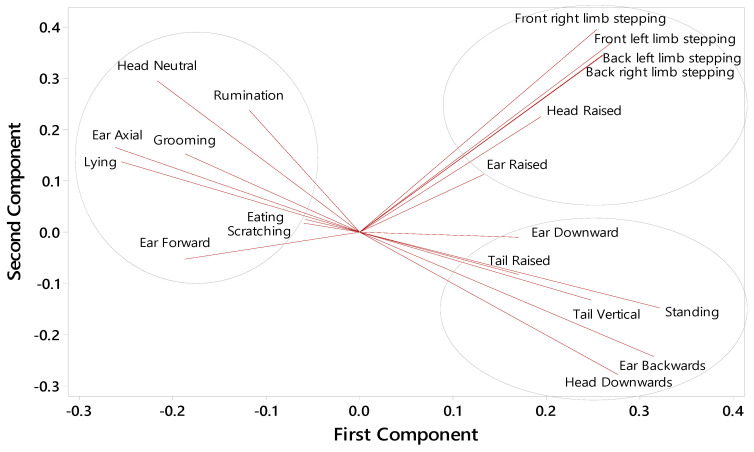
First and second components in a principle component analysis of behaviour in beef cattle during hot conditions.

In relation to the similarity of different behavioural responses to hot conditions, examined in the PCA, two principal components were identified with Eigenvalues of 7.27 and 3.76, explaining 34.6 and 17.9% of the variation, respectively (Figure 9). The first component appears to indicate the degree of comfort, with behaviours associated with cattle comfort on the left-hand side and those associated with discomfort on the right-hand side. The second component appears to separate the behaviours associated with discomfort into adaptive responses, shown at the bottom of the graph, and maladaptive stepping responses, shown at the top of the graph. Tail tucking and tail swishing behaviours were rare and not included in the PCA.

Spearman’s rank correlation of behaviours with respiratory parameters (RR and PS) showed that a high respiration rate was positively associated with a downward head position (correlation coefficient (CC) 0.54; *p* = 0.01), backward ear position (CC 0.49; *p* = 0.03), and vertical tail position (CC 0.56; *p* = 0.01). Regarding stepping, RR had positive correlations with stepping by the front left leg (CC 0.47; *p* = 0.037), back left leg (CC 0.46; *p* = 0.04), and front right leg (CC 0.47; *p* = 0.04) but not the back right leg (CC 0.37; *p* = 0.11). A positive association also existed between panting score and downward head position (CC 0.72; *p* ≤ 0.001), backward ear position (CC 0.53; *p* = 0.015), vertical tail position (CC 0.47; *p* = 0.04), as well as a positive correlation with standing (CC 0.48; *p* = 0.03) and stepping with the front right leg (CC 0.53; *p* = 0.01). There was a negative association between the panting score and neutral head position (CC −0.51, *p* = 0.02).

The association of behaviour in the feedlot (mean ambient temperature (T_A_) 28.58 ± 1.05 °C) with their behaviour change from the first thermoneutral period to the HOT period (27.23 ± 1.73 °C), i.e., those that had the largest behavioural responses to the high temperatures, is presented in Table 6. The animals who moved their head downward the most in the HOT period sustained this behaviour longer in the feedlot. The cattle that moved their ears backward the most in the HOT period in the climate-controlled facility spent longer with their head down and ears forward and less time with ears axial in the feedlot. Similarly, the animals that spent more time with vertical tails in the HOT period were most likely to have a tucked tail, lowered head, and forward ears in the feedlot period. The cattle that exhibited the least amount of time ruminating in the HOT period spent the least amount of time with forward ears and tucked and vertical tails in the feedlot period. The cattle that spent the most time standing in the HOT period spent the most time scratching and the least amount of time with their head in the neutral position in the feedlot period.

## 4. Discussion

Hot environmental conditions provoke behavioural and physiological responses to restore thermal balance [12]. Increased respiration, panting, shade seeking, and standing behaviours are indicators of heat stress in cattle [5,9], in addition to stepping behaviour [29] and postural changes in their head [19,30], tail and ear [31,32]; they all have the potential to detect exposure to excessive heat.

### 4.1. Standing and Stepping

Increased standing, previously recorded in response to heat stress [14,33,34], suggests discomfort [10]. Concurrent increased standing and respiratory distress have also been recorded during heat stress [5]; the former being adaptive through increased evaporative and convective heat exchange [33].

Standing and stepping (particularly left-lateralised) increased and lying decreased in cattle on the finisher diet, suggesting that they were more affected by heat than the substituted diet. Although a high-fibre diet increases heat production due to increased microbial fermentation [35,36], thus increasing the risk of heat stress [37], fibre is necessary to maintain optimal rumen pH by stimulating salivation during bolus chewing [38]. Concentrates particularly reduce rumen pH during hot conditions [39,40], increasing stress [41,42]. In extreme cases, cattle become lame, ataxic, and uncoordinated [43,44,45].

Increased stepping in the HOT period and the positive association of respiratory distress with stepping behaviour during hot days probably also reflects discomfort, as seen in cattle and sheep during other stressful conditions (handling and tick lesions [18], floor movement [46], transport [47], novel stimuli [18,19], hoof lesions [48], and pain [49]). It is unclear whether this is adaptive or not as it increases airflow to the body but may represent an attempt to escape from stressful conditions [18,19].

Cattle respond laterally to novel stimuli [19,50,51]. The right and left legs connect contralaterally with the left and right brain hemispheres, which coordinate proactive and reactive behavioural responses, respectively [52,53]. Stepping proportionately more with left than right legs during the HOT period than the TN and recovery periods suggests stress [52,54], coordinated by the right brain hemisphere, which processes flight-or-fight reactions. The positive association of RR with both front limbs and back left limb stepping confirms the findings by Robins et al. [29] that these limbs are used to express discomfort during stress, with the back right limb acting as a pivot to maintain balance [46].

Animals also stepped more with their back than their front limbs in the HOT period compared to the thermoneutral and recovery periods (Table 4 and Table 5). Hind limbs provide thrust during walking [55] and any escape attempts require locomotion. This could also be related to differential weight distribution between front and back limbs, with more weight on the front legs due to the head [48]. Front limbs are less mobile as they are used for steering and body support [55,56].

### 4.2. Ears

Ear positions reflect emotions [20,31,57], which were negative in our case, with an increase in the backward ear position and a reduction in forward, downward, and axial ear positions during hot conditions. The finisher diet also increased the backward ear position and decreased the axial ear position compared to the substituted diet. Backward ears indicate fear, discomfort, pain, and stress in cattle [31,32]; conversely, forward, downward, and axial ears indicate relaxed cattle [20,32]. Increased back-facing ears probably increase heat retention but may be the result of the activation of the facial, mandibular, and neck muscles during panting. The positive association of respiratory rate during hot days with backward ears supports this explanation. Cattle that moved their ears backward the most in the HOT period lacked persistence in this behaviour, increasing their head down and ears forward the most in the feedlot, suggesting that these measures best depict recovery from hot conditions. A reduction in axial ear positioning and more time with the head oriented downward suggests lethargic and dull behaviour and even depression [20,32]. The return to ears facing forward and axial ears were early indications of recovery from stressful hot conditions [32]. Increased backward ear positioning and decreased axial ear positioning during hot days in cattle on the finisher diet suggest increased heat stress.

### 4.3. Head

Head position not only maintains balance [55,58] but is also linked to emotions [19,32]. Increased downward head movement in the HOT treatment, in the feedlot, and in cattle on the finisher diet indicates discomfort [19,30] and has been observed following castration [20] and handling [30]. The positive association of respiratory distress (RR and PS) with downward head movement supports this and corroborates previous studies on heat stress [1,5,13]. The downward head position may dissipate heat through their trachea better if accompanied by an extension of the neck, or it could be an attempt to inhale cooler air, suggesting that the behaviour is adaptive. Cattle were observed to orient their head downward standing over the water trough (previously reported by Young and Hall [10]) and close to the pen surface, taking advantage of evaporative cooling [10].

### 4.4. Tail

Raised tails, observed more during the HOT period, have been recorded during stress in livestock [19,59]. However, it may also increase the exposure of hairless skin around the anus to aid in evaporative cooling. It was also observed more commonly in cattle on the finisher diet. Tail swishing, reduced in the HOT period, indicates relaxation during brushing and feeding [20], but its reduction may also reduce energy utilisation and heat generation [60] and increase the likelihood of a vertical tail position, which was observed in this study. Stationary vertical tails are also observed during walking, standing, and lying and indicate a calm and relaxed animal [19,20]. However, the association of respiratory distress with a hanging tail in a vertical position confirms its role in the response to heat stress, perhaps to reduce energy utilisation. Notably, the cattle that spent the most time with vertical tails in the HOT period were more likely to have a tucked tail, lowered head, and forward ears in the feedlot, evidence of depression due to their experiences in the hot conditions.

### 4.5. Oral Behaviours

#### 4.5.1. Nutritional Behaviours

A significant decline in the proportion of time spent eating, chewing while eating, ruminating, and dry matter intake were recorded during the HOT period, recovering afterward. Feed intake is responsible for approximately 3 to 8% of cattle heat production [61]. Hot conditions initiate energy-demanding processes to dissipate heat from the body [4], decreasing the amount of energy available for normal behavioural and physiological activities.

Reduced feed intake, eating, chewing while eating [62,63], and rumination [10,64,65] reduce heat produced by digestion [66]. Heat is generated while eating, chewing, and swallowing food, while food moves down the digestive tract (i.e., digestion, including synthesising enzymes), and during excretion. The higher rumination time (Figure 6), eating time, and chewing rate (Figure 7) on most days for cattle receiving a substituted diet can be attributed to the increased fibre intake from lucerne [38]. The cattle that reduced their rumination time the most in the HOT period spent the least amount of time with forward ears and tucked and vertical tails in the feedlot, suggesting that temporarily reduced rumination was an important part of the behavioural responses to hot conditions. The initiation of heat-dissipating mechanisms during hot conditions therefore switches energy from routine behavioural activities, such as eating, ruminating, and self-grooming [60], to other activities, such as increased sweating, panting, and respiration rate [5,66]. These adaptive responses seek to reorient the animal’s behaviour to achieve homeostasis. 

#### 4.5.2. Grooming

Self-grooming declined during the heat load, perhaps due to declining body energy resources in dairy cows [67] and calves [68]. This has been recorded in dairy cows with experimentally induced mastitis and pyrexia [69]. Self-grooming increases water and salt loss through saliva [70]; hence, it was reduced in the HOT period.

#### 4.5.3. Respiration

Increased RR and PS indicate stress as cattle attempt to maintain homeostasis by dissipating the excessive heat load [1]. Respiration rate (RR) and PS increased in animals on a substituted diet, probably due to increased heat production, putting animals at risk of heat stress [37]. The increases in RR and PS during the HOT period compared to the recovery period are consistent with the findings of earlier studies on the impact of heat load on the respiratory dynamics (RR and PS) of cattle [5,13,14,25,71,72]. Hahn et al. [11] reported an increase in respiration rate by 4.3 breath/min per degree °C rise above the baseline respiration rate (60 breaths /min) at the threshold temperature of 21.3 °C. Brown-Brandl et al., [14,73] and Mader et al. [2] also found an increasing trend in the respiration rate and panting score of the feedlot cattle during high ambient temperatures. This increase in respiration rate reflects the imbalance between the heat accumulated and dissipated in heat-stressed animals [73]. To cope with high environmental temperatures, the respiration rate of cattle may go up to 200 breaths per minute during excessive heat load conditions [11]. Increased respiration helps to dissipate approximately 30% of the total heat dissipated, being primarily influenced by changes in ambient temperature, with a minimum lag time of 1–2 h [5,13].

Cattle in heat load conditions spent more time standing, with increased stepping, downward head positioning, backward ear positioning, and raised or vertical tail positioning. Lateralised stepping responses in feedlot cattle appeared to reflect discomfort during the hot environmental conditions, with the forelimbs playing a key role in maintaining the body’s balance. The heat load conditions also reduced eating, chewing, ruminating, grooming, and scratching, probably due to lower energy levels in cattle [67] and calves [68].

Recovery from the heat brought improvement in eating, chewing, ruminating, grooming, and scratching. Most behaviours were potentially adaptive responses to the heat, including increased respiration, panting, standing, nutritional behaviours, grooming, and certain changes in key body parts, backward ears, lowered head and vertical or raised tails, but increased stepping during the HOT period, and behaviour in the feedlot, tucked tail, lowered head and forward ears, appeared to just reflect irritation and/or depression and may not be adaptive. Cattle on a substituted diet with more forage coped better in the heat than a finisher diet, displaying more axial ears and their head in a neutral position, whereas those on the finisher diet were more likely to display more standing, backward ears, and a vertical tail during heat stress. 

## 5. Limitations

The absence of a control group could be a confounding factor related to adaptation and or acclimation to heat stress in beef cattle, i.e., Black Angus. The environmental conditions in the climate-controlled facility were well controlled, and each animal was kept in a separate pen, however, in the feedlot, the environmental conditions were not controlled, and all the animals were kept in a feedlot pen at the end of the experiment. It is possible that some behaviours may have been affected because of these differences in animal management in the feedlot and climate-controlled facility.

A team of five trained observers recorded the respiration rate and panting score (PS), with only one observer recording measurements at any one time, as discussed earlier (Table 3), where inter-observer and or intra-observer metrics were not calculated and should be considered as one of the limitations of this study.

As mentioned earlier, not all behaviours could always be observed: accurate recording of the ear position was not possible when the animal was ruminating, eating, drinking, scratching, or grooming; recording of the head position was not possible during eating, drinking, grooming, or scratching, and recording of the tail position was not possible during defecation, urination, eating, drinking, grooming, or scratching. The inability to record some behaviours when the cattle were in specific positions was a limitation but was not considered to bias treatment effects. In addition, the recording methods used may have affected the results of our study.

A weakness of this study is the lack of physiological indicators (i.e., non-behavioural indicators) of the short-term impact of the heat load period and the capacity of the animal to return to a normal state after the heat load is removed. Indeed, coping with heat challenges is complex and cannot be reduced to a single physiological or behavioural parameter. Nonetheless, commonly used physiological indicators include return to normal body temperature, daily feed intake, or growth rate; however, the dynamics of these variables are not reported in this manuscript. In the absence of these non-behavioural measures, respiratory rate and panting score are recommended as the “gold standard” for the interpretation of the significance of changes in other behaviours.

Some of these behavioural responses can be recorded using surveillance cameras; however, this may be difficult in feedlots. Alternatively, stockpersons can be trained to identify heat-stressed cattle, especially through increased standing time and stepping behaviour, lowered head positioning, backward ear positioning, and panting. The recording of behaviours can be time-consuming and exhausting for observers in hot conditions, especially in a large group of animals. Automated behavioural recordings can be a good replacement. In the future, cattle behavioural responses to heat stress may be detected by remote monitoring equipment, such as remote sensing devices, GPS collars, and pedometers [74,75,76].

## 6. Conclusions

A lowered head, a back-facing ear, a vertical or raised tail, and increased respiration rate and panting persisted during the heat load period, suggesting that these behaviours have diagnostic value. We conclude that cattle have subtle changes in behaviour in response to hot conditions, which could be used to detect the animals that are most affected. We also conclude that some, but not all, responses appear to be adaptive and that changing to a substituted diet with relatively higher forage can reduce hyperthermic behavioural responses.

## Figures and Tables

**Table 1:** The ambient temperature and relative humidity for finisher and substituted cohorts of cattle (*n* = 12) when in the climate-controlled facility.

Day	Treatment Phase	Min T_A_ (°C)	Max T_A_ (°C)	Mean T_A_ (°C)	Min RH (%)	Max RH (%)	Mean RH (%)	Min THI	Max THI	Mean THI
0	ACC	19.7	21.0	20.1	60.9	90.6	66.2	65.5	69.1	66.3
1	ACC	19.7	21.0	20.1	60.9	90.6	66.2	65.5	69.1	66.3
2	ACC	19.5	21.6	20.0	60.0	89.3	67.1	65.3	68.9	66.2
3	TN	19.5	20.8	19.9	61.3	90.1	67.9	65.3	68.8	66.1
4	TN	19.6	24.0	20.2	60.0	89.1	68.2	65.5	72.0	66.4
5	TP1	19.9	40.5	33.2	42.9	88.4	66.1	66.3	92.6	84.9
Finisher Dietary Cohort: Transition to 30 °C from 00:00 h on day 5 Substituted Dietary Cohort: Transition to 30 °C from 21:00 h on day 5										
6	HOT	28.4	40.2	33.0	43.3	82.8	65.8	80.5	91.8	84.8
7	HOT	28.4	38.1	32.1	42.3	84.2	63.7	78.3	89.1	83.0
8	HOT	24.9	34.3	28.7	44.3	82.0	65.9	73.6	85.0	78.4
9	HOT	22.6	34.4	28.0	45.81	79.5	66.2	69.9	85.7	77.5
10	HOT	20.6	30.3	24.3	54.4	80.5	66.7	67.2	80.0	72.3
11	HOT	20.4	30.4	24.2	45.3	80.6	65.8	67.1	79.2	72.0
12	HOT	19.7	21.3	20.3	50.0	90.5	64.6	65.8	68.8	66.4
13	TP2	19.7	20.7	20.1	56.4	91.3	65.5	65.6	68.6	66.2
14	Recovery	19.7	21.4	20.1	58.1	89.0	66.7	65.6	69.6	66.2
15	Recovery	19.6	20.5	19.9	58.4	90.3	66.4	65.6	68.2	66.0
16	Recovery	19.4	25.0	20.5	57.8	93.5	66.4	65.2	73.2	66.8
17	Recovery	19.3	23.7	21.1	58.1	69.0	61.9	64.9	71.1	67.5

T_A_: ambient temperature (°C); RH: relative humidity; THI: temperature–humidity index; ACC: acclimatisation to climate-controlled facility; TN: thermoneutral conditions before high-temperature treatment; TP1 and TP2: transition phases to and from hot conditions; HOT: high-temperature treatment; and recovery: thermoneutral conditions after high-temperature treatment as a recovery period.

**Table 2:** Diet ingredients and nutrient composition for starter, intermediate, finisher, and substituted diets fed to cattle.

Item	Starter	Intermediate	Finisher	Substituted
**Ingredients, % of diet**				
Grain mix *	62.1	74.5	86.8	78.7
Whole cottonseed	9.0	16.5	9.0	9.0
Lucerne hay	28.9	9.0	4.2	12.3
**Nutrient composition**				
DM, g/kg fresh weight	880	893	887	886
ADF, g/kg DM	263	257	119	177
NDF, g/kg DM	404	375	229	253
NE_g_, MJ/kg DM	29	29	30	30
ME, MJ/kg DM	116	119	132	131
DE, MJ/kg DM	143	147	163	162
Crude fibre, g/kg DM	218	197	87	124
Nitrogen-free extract, g/kg DM	503	548	678	685
Fat, g/kg DM	46	43	46	43
Feed digestibility, g/kg DM	768	791	861	868
Digestible DM, g/kg DM	676	707	763	769
Digestible protein g/kg DM	133	125	130	131
Starch, g/kg DM	229	218	432	432

* Grain mix: 9.2% feedlot pellet, 89.2% steam-rolled barley, and 1.6% vegetable oil. The feedlot pellet contained 55.9% milled wheat, 2.6% ammonium sulfate, 12.5% rolled wheat, 15.6% calcium carbonate, 100 0.3% Rumensin, 0.7% magnesium oxide, 0.34% zinc supplement (Availa zinc 100), 3.1% vegetable oil, 2.8% NaCl, 5.7% urea, 500 0.009% vitamin A, 0.057% vitamin E, and 0.385% mineral supplement (XFE-Select L).

**Table 3:** Ethogram for recorded behaviours for cattle (n = 24) housed in individual pens in the climate-controlled facility.

Item	Description
Respiration rate	Time taken for 10 breaths, determined by flank movement
Panting score	Animal visually scored for the extent of panting based on a score scale of 0 to 4.5
Standing	Animal standing with limb positioned upright
Lying	Animal resting on the floor with their limb laterally or sternally recumbent
Eating	Animal consuming feed at the trough
Chews while eating	Chews counted for one minute at the time of morning feed
Rumination	Animal chewing a bolus or regurgitating bolus
Grooming	Animal licking any part of the body or striking one part with another part of the body
Scratching	Animal rubbing or striking any part of the body against the fixture of the pen
Ear positions	
Ear raised	Both ears held upright above the neck with the ear pinnae facing forward or to the side
Ear forward	Both ear pinnae directed forward in front of the focal animal and the ear held horizontally
Ear backward	Both ears being held backward on the focal animal’s head
Ear downward	Both ears being loosely hung downward, falling perpendicular to the head
Ear specific	Both ear pinnae (right and left) oriented in the opposite direction or perpendicular to the head rump axis, failing to satisfy raised, forward, backward, and downward ear positions
Head positions	
Head raised	The head held upright above the withers or the body’s topline
Head neutral	The head held horizontally at the level of the withers or the body’s topline
Head downward	The head held downward below the withers or the body’s topline
Stepping	
Front right (FR) limb	Animal raising a front right limb and replacing it forthwith on the surface of the pen
Front left (FL) limb	Animal raising a front left limb and replacing it forthwith on the surface of the pen
Back right (BR) limb	Animal raising a back right limb and replacing it forthwith on the surface of the pen
Back left (BL) limb	Animal raising a back left limb and replacing it forthwith on the surface of the pen
Tail positions	
Tail raised	Tail held in a fixed position, held at 45 degrees from the vertical position
Tail vertical	Tail hanging downward from the vertical line of the body and is vertical with no movements
Tail swishing	Swift movement of the tail in any direction around the hind quarters from its base in a side-to-side flicking manner
Tail tucked	Tail held tightly pressed in a fixed position against the rump, with the tip of the tail tucked behind the hind limb

Adapted from Idris [22] and Goma et al. [19].

**Table 4:** Behavioural and physiological measurements in cattle (n = 20) receiving a finisher or substituted diet during the initial thermoneutral period (TN) and when exposed to high temperatures (HOT).

Behaviour	Finisher Diet		Substituted Diet		SED	F-Value (1, 18 d.f. ^†^)	*p*-Value	
	TN	HOT	TN	HOT			Period (P)	D × P
**Stepping**								
FR limb, Log_10_ + 1 counts/5 min	0.69	0.79	0.70	0.79				
(counts/5 min)	(3.90)	(5.17)	(4.01)	(5.17)	0.0074	331.35	≤0.001	0.15
FL limb, Log_10_ + 1 counts/5 min	0.73	0.84	0.68	0.81				
(counts/5 min)	(4.37)	(5.92)	(3.79)	(5.46)	0.0103	276.25	≤0.001	0.51
BR limb, Log_10_ + 1 counts/5 min	0.81	1.03	0.71	0.97				
(counts/5 min)	(5.46)	(9.72)	(4.13)	(8.33)	0.023	229.66	≤0.001	0.24
BL limb, Log_10_ + 1 counts/5 min	0.84	1.06	0.71	0.99				
(counts/5 min)	(5.92)	(10.48)	(4.13)	(8.77)	0.025	200.85	≤0.001	0.17
Total stepping, Log_10_ + 1 counts/5 min	1.31	1.51	1.23	1.46				
(counts/5 min)	(19.42)	(31.36)	(15.98)	(27.84)	0.0198	233.90	≤0.001	0.37
R/L limb, ratio of Log_10_ + 1 counts/5 min	0.28	0.28	0.31	0.29				
(ratio of counts/5 min)	(0.91)	(0.91)	(1.04)	(0.95)	0.0158	6.73	0.02	0.064
F/B limb, ratio of Log_10_ + 1 counts/5 min	0.24	0.19	0.28	0.21				
(ratio of counts/5 min)	(0.74)	(0.55)	(0.91)	(0.62)	0.0112	57.88	≤0.001	0.24
**Standing/lying**								
Standing, Log_10_ + 1 prop. time	0.14	0.17	0.15	0.17				
(prop. time)	(0.38)	(0.48)	(0.41)	(0.48)	0.00202	254.50	≤0.001	0.075
Lying, Log_10_ + 1 prop. time	0.21	0.18	0.20	0.18				
(prop. time)	(0.62)	(0.51)	(0.59)	(0.51)	0.0012	836	≤0.001	0.71
**Ears, head, and tail**								
Ear backward, Log_10_ + 1 prop. time	0.088 ^c^	0.23 ^a^	0.0501 ^d^	0.17 ^b^				
(prop. time)	(0.22)	(0.71)	(0.12)	(0.49)	0.00242	6108.09	≤0.001	≤0.001
Ear forward, Log_10_ + 1 prop. time	0.11	0.0396	0.11	0.047				
(prop. time)	(0.29)	(0.095)	(0.29)	(0.12)	0.00488	386.44	≤0.001	0.24
Ear axial, Log_10_ + 1 prop. time	0.078 ^b^	0.0304 ^c^	0.11 ^a^	0.094 ^a^				
(prop. time)	(0.19)	(0.073)	(0.276)	(0.24)	0.00389	116.37	≤0.001	≤0.001
Head downward, Log_10_ + 1 prop. time	0.014 ^c^	0.13 ^a^	0.016 ^c^	0.099 ^b^				
(prop. time)	(0.033)	(0.34)	(0.037)	(0.26)	0.00716	387.1	≤0.001	0.01
Head neutral, Log_10_ + 1 prop. time	0.28	0.21	0.27	0.22				
(prop. time)	(0.88)	(0.63)	(0.88)	(0.66)	0.0049	272.19	≤0.001	0.096
Tail vertical, Log_10_ + 1 prop. time	0.27 ^c^	0.29 ^a^	0.27 ^c^	0.28 ^b^				
(prop. time)	(0.86)	(0.96)	(0.85)	(0.92)	0.00181	227.66 (1, 8)	≤0.001	0.002
Tail swishing, Log_10_ + 1 prop. time	0.000157	0.000629	0.000846	0.000646				
(prop. time)	(0.000362)	(0.00145)	(0.00195)	(0.00149)	0.0000505	0.14	0.71	0.36
**Oral behaviours**								
Groom, Log_10_ + 1 prop. time	0.0081	0.0017	0.011	0.0055				
(prop. time)	(0.019)	(0.00397)	(0.29)	(0.013)	0.000335	629.29	≤0.001	0.14
Scratch, Log_10_ + 1 prop. time	0.0047	0.00085	0.0056	0.0018				
(prop. time)	(0.011)	(0.0019)	(0.013)	(0.00404)	0.000492	120.87	≤0.001	0.99
Rumination, Log_10_ + 1 prop. time	0.067 ^a^	0.016 ^c^	0.066 ^a^	0.026 ^b^				
(prop. time)	(0.17)	(0.038)	(0.16)	(0.061)	0.00181	1260.63	≤0.001	0.001
Eating, Log_10_ + 1 prop. time	0.015	0.0041	0.017	0.0067				
(prop. time)	(0.034)	(0.0094)	(0.039)	(0.016)	0.000894	318.49	≤0.001	0.55
Chewing while eating, Log_10_ + 1 chews/minute	0.067 ^a^	0.016 ^c^	0.066 ^a^	0.026 ^b^				
(chews/minute)	(0.17)	(0.038)	(0.16)	(0.061)	0.00181	1260.63	≤0.001	0.001
Dry matter intake, Log_10_ + 1 kg/day	1.05	0.79	1.08	0.83				
(kg/day)	(10.22)	(5.17)	(11.02)	(5.76)	0.0312	135.32	≤0.001	0.94
Respiration rate, Log_10_ + 1 breaths/min	1.77	2.06	1.80	2.07				
(breaths/min)	(57.88)	(113.82)	(62.1)	(116.49)	0.00716	2939.5	≤0.001	0.055
Panting score (PS), Log_10_ + 1 PS score	0.31 ^b^	0.44 ^a^	0.34 ^b^	0.44 ^a^				
(PS score)	(1.04)	(1.75)	(1.19)	(1.75)	0.00716	547.79	≤0.001	0.04

FR, front right limb stepping; FL, front left limb stepping; BL, back left limb stepping; BR, back right limb stepping; Log_10_ + 1, logbase_10_ + 1; R/L, right/left limb stepping; F/B, front/back limb stepping; SED, standard error of the difference between two means; HOT, high-temperature treatment period (days 6–12); TN, thermoneutral period before high-temperature treatment (day 3); ^†^ treatment, error degrees of freedom; D, diet; and P, period. Means with different superscripts within rows differ significantly by Fisher’s test.

**Table 5:** Behavioural and physiological measurements in cattle (n = 20) receiving a finisher or substituted diet during the high-temperature (HOT) period and in the thermoneutral recovery period using the TN period as a covariate (Cov).

Behaviour	Finisher Diet		Substituted Diet		SED	F-Value (d.f. ^†^)	*p*-Value			
	HOT	Recovery	HOT	Recovery			Period (P)	Diet (D)	D × P	D × d
**Stepping**										
FR limb, Log_10_ + 1 counts/5 min	0.81	0.71	0.70	0.52						
(counts/5 min)	(5.51)	(4.07)	(4.02)	(2.29)	0.061	47.69 (1, 162)	≤0.001	0.03	0.08	≤0.001
FL limb, Log_10_ + 1 counts/5 min	0.84 ^a^	0.71 ^a^	0.73 ^ab^	0.50 ^c^						
(counts/5 min)	(5.90)	(4.07)	(4.46)	(2.15)	0.064	71.17 (1, 162)	≤0.001	0.006	0.02	≤0.001
BR limb, Log_10_ + 1 counts/5 min	0.98 ^a^	0.86 ^b^	0.91 ^ab^	0.64 ^c^						
(counts/5 min)	(8.58)	(6.25)	(7.06)	(3.32)	0.077	54.54 (1, 162)	≤0.001	0.02	0.005	≤0.001
BL limb, Log_10_ + 1 counts/5 min	1.04	0.85	0.91	0.64						
(counts/5 min)	(9.91)	(6.03)	(7.10)	(3.39)	0.073	82.07 (1, 162)	≤0.001	0.01	0.142	≤0.001
Total stepping, Log_10_ + 1 counts/5 min	1.50 ^a^	1.33 ^b^	1.36 ^ab^	1.07 ^c^						
(counts/5 min)	(30.37)	(20.38)	(22.14)	(10.85)	0.076	74.46 (1, 162)	≤0.001	0.006	0.02	≤0.001
R/L limb, ratio of Log_10_ + 1 counts/5 min	0.28	0.31	0.30	0.32						
(ratio of counts/5 min)	(0.89)	(1.02)	(0.98)	(1.07)	0.014	27.31 (1, 162)	≤0.001	0.04	0.28	≤0.001
F/B limb, ratio of Log_10_ + 1 counts/5 min	0.20	0.22	0.23	0.25						
(ratio of counts/5 min)	(0.58)	(0.65)	(0.68)	(0.77)	0.023	6.55 (1, 162)	0.01	0.07	0.79	0.22
**Standing/lying**										
Standing, Log_10_ + 1 prop. time	0.18	0.18	0.15	0.13						
(prop. time)	(0.52)	(0.51)	(0.42)	(0.36)	0.014	4.27 (1, 162)	0.04	0.01	0.14	0.07
Lying, Log_10_ + 1 prop. time	0.17 ^b^	0.16 ^b^	0.19 ^b^	0.21 ^a^						
(prop. time)	(0.47)	(0.46)	(0.54)	(0.61)	0.014	2.96 (1, 162)	0.09	0.03	0.01	0.03
**Ears, head, and tail**										
Ear backward, Log_10_ + 1 prop. time	0.24 ^a^	0.064 ^c^	0.16 ^b^	0.066 ^c^						
(prop. time)	(0.72)	(0.160)	(0.45)	(0.164)	0.0188	423 (1, 162)	≤0.001	≤0.001	≤0.001	≤0.001
Ear forward, Log_10_ + 1 prop. time	0.038 ^b^	0.087 ^a^	0.047 ^b^	0.077 ^a^						
(prop. time)	(0.091)	(0.223)	(0.115)	(0.194)	0.0112	104.71 (1, 162)	≤0.001	0.94	0.01	0.03
Ear axial, Log_10_ + 1 prop. time	0.024 ^c^	0.13 ^a^	0.095 ^b^	0.12 ^a^						
(prop. time)	(0.057)	(0.34)	(0.245)	(0.32)	0.0155	140 (1, 162)	≤0.001	0.01	≤0.001	≤0.001
Head downward, Log_10_ + 1 prop. time	0.134 ^a^	0.037 ^c^	0.085 ^b^	0.037 ^c^						
(prop. time)	(0.360)	(0.090)	(0.22)	(0.088)	0.0189	123 (1, 162)	≤0.001	0.03	≤0.001	0.002
Head neutral, Log_10_ + 1 prop. time	0.20 ^c^	0.25 ^a^	0.23 ^b^	0.24 ^ab^						
(prop. time)	(0.59)	(0.79)	(0.68)	(0.74)	0.018	29.43 (1, 162)	≤0.001	0.54	0.006	0.01
Tail vertical, Log_10_ + 1 prop. time	0.296	0.270	0.278	0.251						
(prop. time)	(0.978)	(0.864)	(0.898)	(0.782)	0.0124	38.70 (1, 162)	≤0.001	0.008	0.86	0.30
Tail swishing, Log_10_ + 1 prop. time	0.00088	0.0071	0.00039	0.0034						
(prop. time)	(0.0020)	(0.017)	(0.00089)	(0.0078)	0.00649	4.21 (1, 179)	0.042	0.35	0.47	0.84
**Oral behaviours**										
Groom, Log_10_ + 1 prop. time	0.0023 ^c^	0.016 ^a^	0.0046 ^c^	0.011 ^b^						
(prop. time)	(0.0053)	(0.038)	(0.011)	(0.026)	0.00246	140.71 (1, 161)	≤0.001	0.295	≤0.001	0.002
Scratch, Log_10_ + 1 prop. time	0.00093 ^c^	0.012 ^a^	0.0015 ^c^	0.0066 ^b^						
(prop. time)	(0.0021)	(0.028)	(0.0035)	(0.015)	0.00197	132 (1, 157)	≤0.001	0.038	≤0.001	0.90
Rumination, Log_10_ + 1 prop. time	0.012 ^d^	0.059 ^a^	0.029 ^c^	0.047 ^b^						
(prop. time)	(0.029)	(0.145)	(0.067)	(0.113)	0.010	85.21 (1, 162)	≤0.001	0.60	≤0.001	0.04
Eating, Log_10_ + 1 prop. time	0.0035	0.0075	0.0069	0.012						
(prop. time)	(0.0081)	(0.017)	(0.016)	(0.028)	0.00411	9.57 (1, 162)	0.002	0.06	0.80	0.05
Chewing while eating, Log_10_ + 1 chews/minute	1.85	1.97	1.87	1.97						
(chews/minute)	(70.21)	(91.70)	(73.32)	(92.28)	0.0155	373.86 (1, 128)	≤0.001	0.19	0.15	0.05
Dry matter intake, Log_10_ + 1 kg/day	0.76	0.95	0.80	0.98						
(kg/day)	(4.80)	(7.98)	(5.28)	(8.45)	0.045	140.69 (1, 162)	≤0.001	0.36	0.70	0.75
Respiration rate, Log_10_ + 1 breaths/min	2.04 ^a^	1.66 ^c^	2.05 ^a^	1.73 ^b^						
(breaths/min)	(108.27)	(44.19)	(111.61)	(52.70)	0.0441	6923.51 (1, 3810.59)	≤0.001	0.02	≤0.001	≤0.001
Panting score (PS), Log_10_ + 1 PS score	0.44 ^a^	0.20 ^c^	0.44 ^a^	0.25 ^b^		3882.21				
(PS score)	(1.74)	(0.57)	(1.75)	(0.76)	0.036	(1, 3809.74)	≤0.001	0.02	≤0.001	≤0.001

FR, front right limb stepping; FL, front left limb stepping; BL, back left limb stepping; BR, back right limb stepping; Log_10_ + 1, logbase_10_ + 1; R/L, right/left limb stepping; F/B, front/back limb stepping; SED, standard error of the difference between two means; HOT, high-temperature treatment period (days 6–12); recovery thermoneutral period after high-temperature treatment (days 14–16); ^†^ treatment, error degrees of freedom; D, diet; D, day, and P: period. Means with different superscripts within rows differ significantly by Fisher’s test.

**Table 6:** Significant Spearman’s correlations between behaviour responses of cattle to thermal stress (HOT period minus TN period) and their behaviour in the feedlot.

Behaviour in the Hot Period–Prior TN Period	Differential Mean ± SE (Prop. of Time) for the 2 Periods	Feedlot Behaviour	Correlation Coefficient	*p*-Value
Head downward	0.26 ± 0.02	Head downward	0.537	0.015
Ear backward	0.43 ± 0.02	Head downward	0.704	0.001
	0.43 ± 0.02	Ear forward	0.694	0.001
	0.43 ± 0.02	Ear axial	−0.699	0.001
Tail vertical	0.084 ± 0.007	Tail tucked	0.530	0.016
	0.084 ± 0.007	Ear forward	0.592	0.006
	0.084 ± 0.007	Head downward	0.689	0.001
Rumination	−0.12 ± 0.004	Ear forward	−0.581	0.007
	−0.12 ± 0.004	Tail tucked	−0.605	0.005
	−0.12 ± 0.004	Tail vertical	−0.554	0.011
Standing	0.076 ± 0.005	Scratching	0.561	0.010
	0.076 ± 0.005	Head neutral	−0.537	0.015

## Data Availability

Data is available on request from the corresponding author.
